# Letter to the Editor on “Causal associations between prostate diseases, renal diseases, renal function, and erectile dysfunction risk: a 2-sample Mendelian randomization study”

**DOI:** 10.1093/sexmed/qfae058

**Published:** 2024-09-15

**Authors:** Youqian Zhang, Li Li, Qiong Wen

**Affiliations:** Department of Oncology, First Affiliated Hospital of Yangtze University, Jingzhou, Hubei Province, 434000, China; Department of Oncology, First Affiliated Hospital of Yangtze University, Jingzhou, Hubei Province, 434000, China; Department of Oncology, First Affiliated Hospital of Yangtze University, Jingzhou, Hubei Province, 434000, China

Urinary system disorders interact with erectile dysfunction (ED) through diverse mechanisms, from hemodynamic changes to neuroendocrine regulation. Recently, Dilixiati et al^1^ explored the causal links between prostate and renal diseases with ED in a Mendelian randomization (MR) study. We commend the authors’ efforts but note that dataset selection, sample overlap, and pleiotropy may compromise the findings’ robustness; we have several suggestions to share.

MR analyses have become popular for evidencing potential causal relationships. Sample overlap is a key factor in MR analyses, moderating biases such as weak instrument bias and winner’s curse. Using the same individuals for genetic variants, exposures, and outcomes exaggerates significance and boosts type I error rates, known as “sample overlap bias,” escalating with overlap degree.^2^ Notably, the authors included a meta-analysis of 3 cohorts in their data selection on ED, showing substantial overlap, especially within the UK Biobank cohort (Table 1). Therefore, we recommend using MRLap, which employs cross-trait linkage disequilibrium score regression (LDSC) intercepts to correct biases from sample overlap and the winner’s curse, ensuring robust results.^3^

**Table 1:** Summary of sample overlap rate calculations.

**Phenotype**	**Sample size**	**Overlapping study**	**Sample overlap (%)**
Prostate cancer	140 254	NA	0
Hyperplasia of prostate	85 917	NA	0
Chronic kidney disease	117 165	EGCUT	9 (1780/17 969)
IgA nephropathy	477 784	UKB	59 (202 402/344 365)
Membranous nephropathy	7979	NA	0
Nephrotic syndrome	215 099	NA	0
Calculus of kidney and ureter	218 414	NA	0
Microalbumin in urine	108 706	UKB	54 (108 706/202 402)
Urinary albumin excretion	382 500	UKB	53 (202 402/382 500)
Potassium in urine	395 995	UKB	51 (202 402/395 995)
Creatinine (enzymatic) in urine	327 525	UKB	62 (202 402/327 525)
Sodium in urine	326 831	UKB	62 (202 402/326 831)
Serum creatinine (eGFRcrea)	133 814	EGCUT	15 (2643/17 969)
Serum cystatin C (eGFRcys)	33 152	EGCUT	6 (1037/17 969)
Kidney injury molecule 1 levels	21 758	NA	0

Values are % (n/n), unless otherwise indicated.

Abbreviations: ED, erectile dysfunction; EGCUT, Estonian Genome Center of the University of Tartu; NA, not available; UKB, UK Biobank.

Samples from a single database may not adequately represent the genetic background and exposure scenarios of European ancestries. The original analysis relied solely on 1 database, which could potentially compromise its robustness. Therefore, using FinnGen or the UK Biobank as replication cohorts to validate findings without sample overlap and conducting a meta-analysis with the discovery cohort’s effect sizes could mitigate the inherent selection biases of single samples. Additionally, the exposure data were sourced from the outdated FinnGen Release 5 (2021), neglecting the more recent Release 10 (2023). Additionally, as the authors noted regarding the interaction between exposure and outcomes, it is necessary to supplement the study with reverse MR analyses. Utilizing the latest datasets, increasing cohort validation, and conducting reverse analyses would beneficially enhance the robustness and generalizability of the research findings.

Given that most genetic variants explain only a small proportion of phenotypic variance, insufficient statistical power remains a significant challenge in MR studies.^4^ This often leads to inaccurate detection of causal effects and increases the risk of false positives. We appreciate that the authors eliminated confounding single nucleotide polymorphisms using the PhenoScanner to uphold the independence assumption and avoid biases due to unrelated horizontal pleiotropy. However, recent studies emphasize that excessive removal can lead to information loss, thereby increasing the risk of type I errors. If single nucleotide polymorphisms related to confounding factors are critical for the phenotypes under study, their exclusion could inadvertently reduce noise, thereby weakening detection capabilities.^5^ Additionally, the bias due to related horizontal pleiotropy was not addressed. According to the latest MR guidelines, incorporating CAUSE (Causal Analysis Using Summary Effect Estimates) analysis and calculations for statistical power would be beneficial in supporting causal conclusions.

In summary, we appreciate the authors’ contributions to understanding the connection between urological diseases and ED. However, in this complex field, precise data and rigorous methodological design are crucial for establishing causal relationships.

## Data Availability

All data are publicly available and can be accessed in the original text of the comments.
