# Correction to: Letter to the Editor on “Causal associations between prostate diseases, renal diseases, renal function, and erectile dysfunction risk: a 2-sample Mendelian randomization study”

**DOI:** 10.1093/sexmed/qfae069

**Published:** 2024-09-24

This is a correction to: Youqian Zhang, Li Li, Qiong Wen, Letter to the Editor on ``Causal associations between prostate diseases, renal diseases, renal function, and erectile dysfunction risk: a 2-sample Mendelian randomization study'', *Sexual Medicine*, Volume 12, Issue 4, August 2024, https://doi.org/10.1093/sexmed/qfae058

In the originally published version of the manuscript, all indications and details for Dr Qiong Wen as co-corresponding author were erroneously omitted. These details are now emended in the article.

